# EXPLORING INTERPROFESSIONAL HEALTH STUDENTS’ ATTITUDES AND PERCEPTIONS OF AGING AND CAREERS IN GERONTOLOGY

**DOI:** 10.1093/geroni/igad104.3329

**Published:** 2023-12-21

**Authors:** Brianna Garrison

**Affiliations:** Southern Connecticut State University, New Haven, Connecticut, United States

## Abstract

ageism continues to impact healthcare professional’s attitudes toward older adults. Older adults are often assumed to be frail, weak, or a burden on society. Addressing ageist attitudes and myths about ageing, which often lead to discrimination, can impact policy decisions. Knowledge of opportunities available to older adults to increase healthy ageing is vital in any healthcare training program. Recognizing the growing needs to prepare health professionals to work with the increasing numbers of diverse older adults, this research survey conducted for the college of health and human sciences (CHHS) to examine the students’ attitudes and perceptions of ageing, interest in ageing careers, knowledge gaps in ageing, and ageing topics of interest. This survey resulted in 141 respondents who helped provide an informed baselines of CHHS student misconceptions about ageing, baseline of learning needs, and topics and practice opportunities of interest. This poster will share the unique results of this survey providing insight into students’ attitudes and perceptions on ageing. Additionally, this poster will provide a brief picture of the college’s response to the survey including curriculum development and systemic changes.

